# Supplementary material for: Chd1 protects genome integrity at promoters to sustain hypertranscription in embryonic stem cells
Source: Nat Commun. 2021 Aug 11;12:4859. doi: 10.1038/s41467-021-25088-3 (PMC8357957; doi:10.1038/s41467-021-25088-3)
Supplement: Supplementary file 1 — Supplementary Information [file 41467_2021_25088_MOESM1_ESM.pdf]

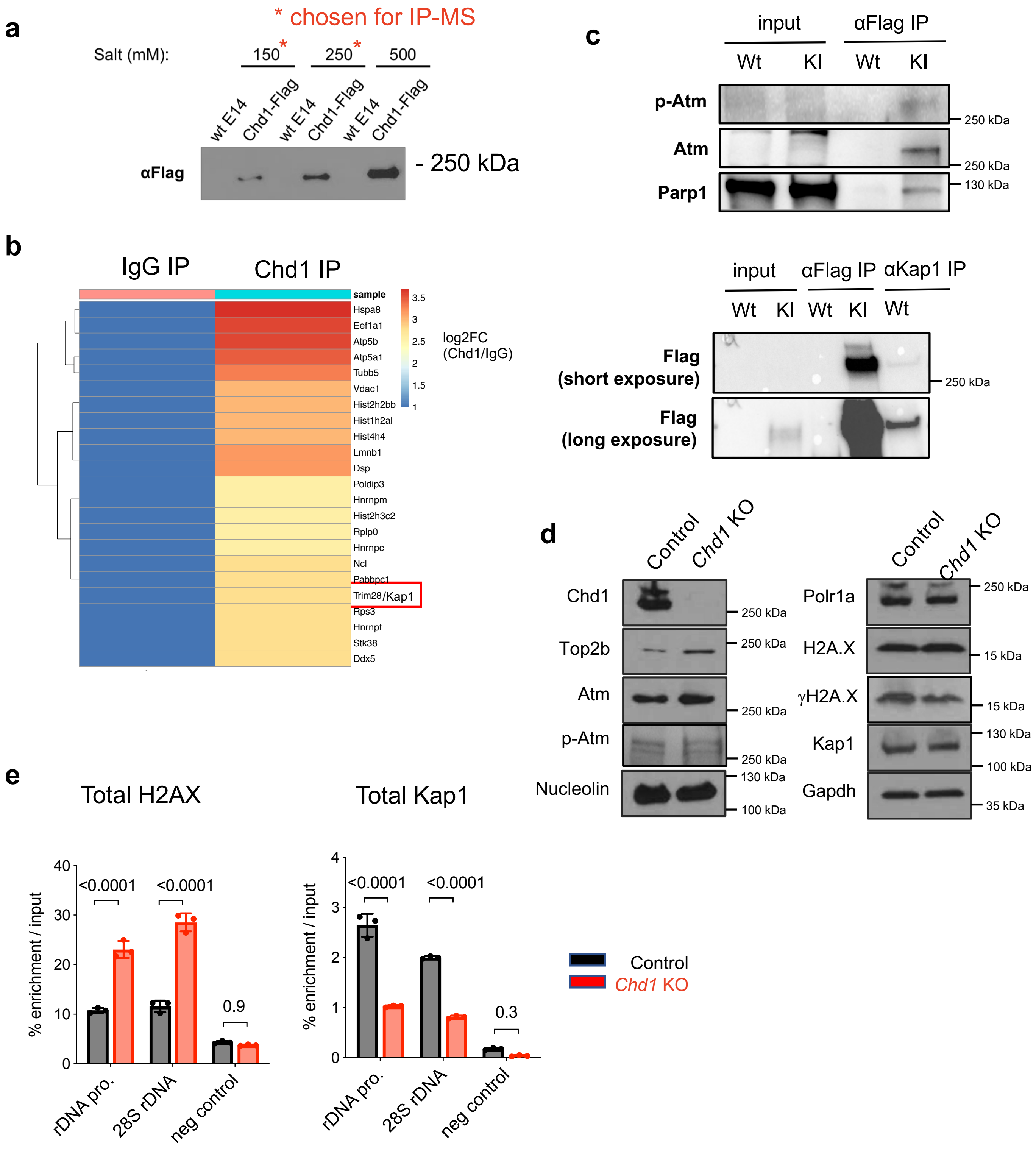

Supplementary Figure 1 Further characterization of Chd1 interactors and protein abundance in *Chd1* KO cells.
A) Western blot showing immunoprecipitated Chd1 levels at increasing salt concentrations. N=2 biologically independent experiments.
B) Results of the 250 mM salt Chd1 IP-MS.
C) Additional Co-IP validation of the interaction between Chd1 and DNA repair factors in ES cells. N=2 biologically independent experiments.
D) Expression levels of indicated proteins in control and *Chd1* KO ES cells. Whole cell extracts were used. N=2 biologically independent experiments.
E) Quantification of total H2A.X and Kap1 levels at rDNA in control and *Chd1* KO cells by ChIP-PCR. N=3 biologically independent experiments. Graphs show mean and standard deviation. Statistical tests performed are two-way ANOVA with Sidak's correction. Values above bars indicate p-values.

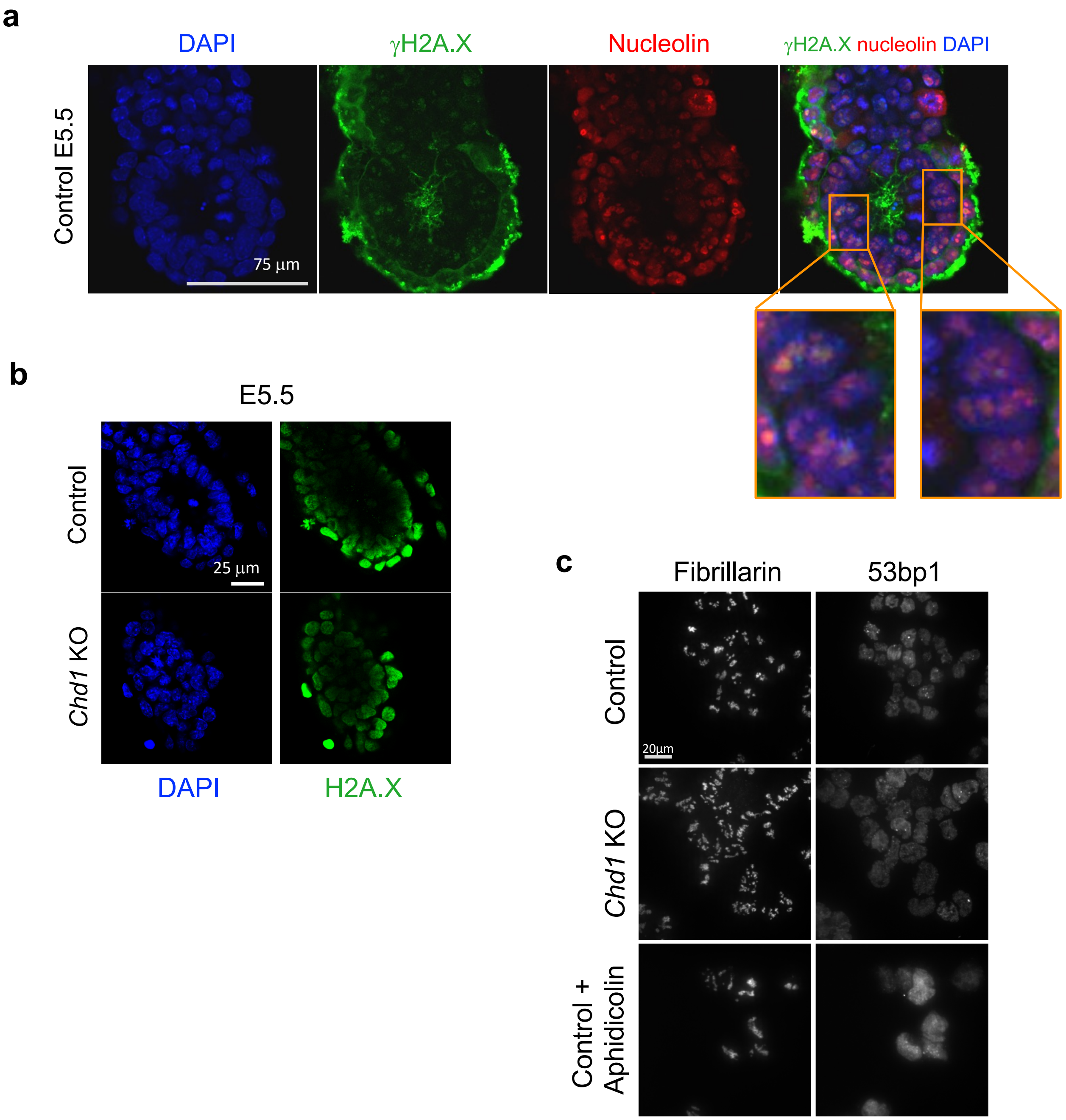

Supplementary Figure 2 Immunofluorescence analyses of DNA damage markers in control and *Chd1* KO cells.

A) γH2A.X co-localizes with nucleolin in control E5.5 embryos. N=4 (minimum) biologically independent embryos.

B) Total H2A.X remains unchanged upon *Chd1* deletion at E5.5. N=4 (minimum) biologically independent embryos.

C) Some foci of 53BP1 foci are detected in both control and *Chd1* KO ES cells, but 53BP1 is robustly induced with exogenous DNA damage (Aphidicolin treatment). N=2 biologically independent experiments.

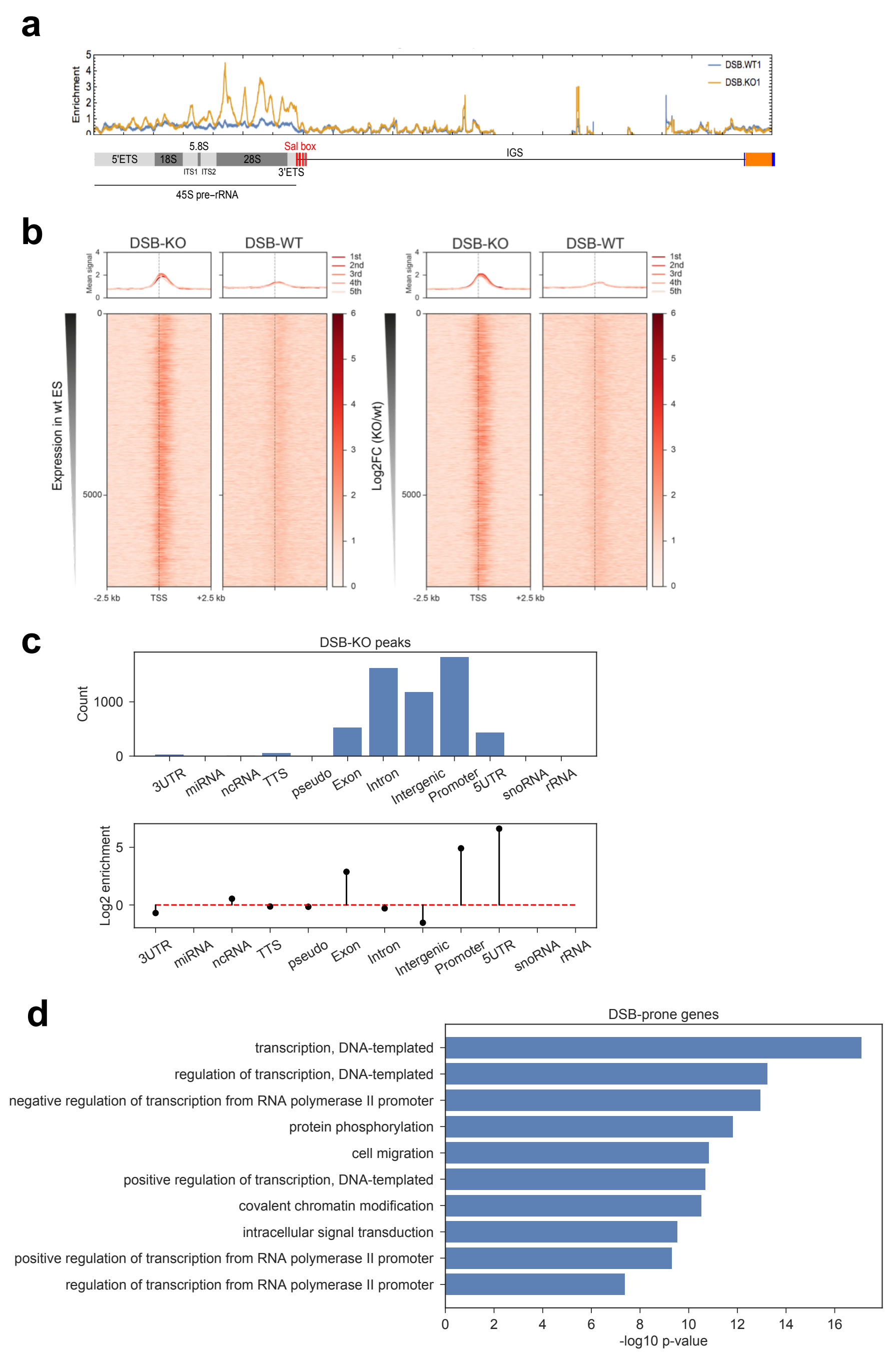

Supplementary Figure 3 Characterization of DSB-prone genomic regions and associated gene functions. A) Mapping of DSB-seq reads to the consensus 45K ribosomal RNA sequence. Schematic shows the sequence elements within the locus.

B) Heatmaps showing DSB levels in wt and *Chd1* KO cells. Genes are ranked based on expression levels in wt cells (left panel) or fold change in *Chd1* KO vs. wt cells (right panel). Only protein-coding genes with unique TSSs and provided expression values from Guzman-Ayala et al.[3] are included.

C) Distribution and enrichment of DSBs on various genomic annotations. The log2 enrichment was calculated as the log2 ratio of the fraction of peaks associated with a specific genomic annotation and the fraction of the genome assigned to the same genomic annotation, as returned by HOMER.

D) Gene ontology pathways associated with 1785 DSB-prone genes. Statistical test used is a modified one-sided Fisher's exact test called EASE score generated by the DAVID software.

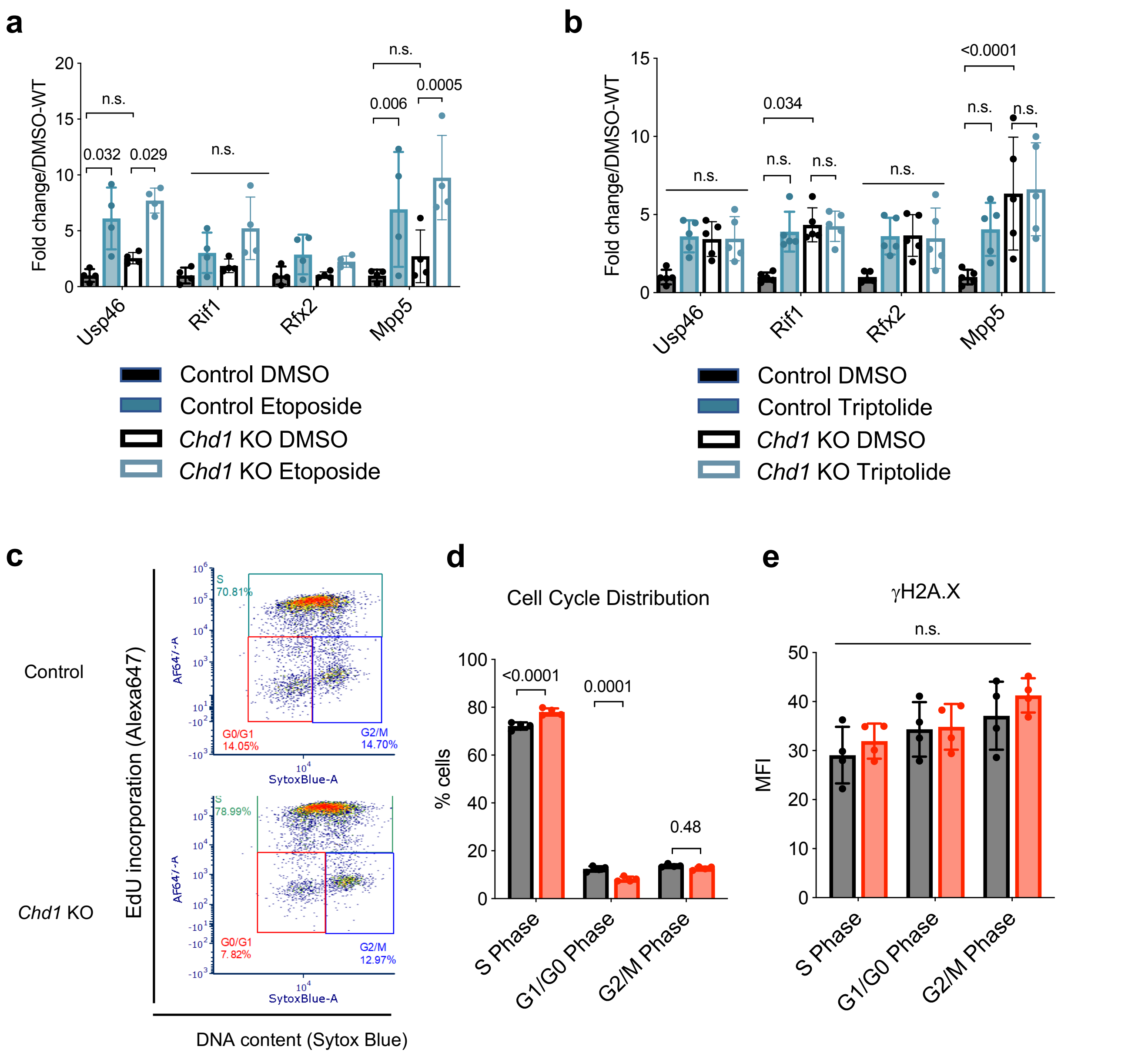

Supplementary Figure 4 Characterization of cell cycle in relation to DNA damage in *Chd1* KO cells.

A) Combined plots of gene promoter DSB levels in control and *Chd1* KO cells with or without treatment of etoposide. Y axis shows fold change over DMSO-treated control (wild-type) cells. N=4 biologically independent experiments. Graphs show mean and standard deviation. Statistical tests performed are two-way ANOVA with Sidak's correction. Values above bars indicate p-values.

B) Combined plots of gene promoter DSB levels in control and *Chd1* KO cells with or without treatment of triptolide. Y axis shows fold change over DMSO-treated control (wild-type) cells. N=5 biologically independent experiments. Graphs show mean and standard deviation. Statistical tests performed are two-way ANOVA with Sidak's correction. Values above bars indicate p-values.

C) Flow cytometry analysis for cell cycle distribution of control and *Chd1* KO cells. Cells were analyzed by EdU incorporation (Alexa 647) and DNA content (Sytox Blue).

D) Cell cycle distribution of control and *Chd1* KO cells. N= 4 biologically independent experiments. Graphs show mean and standard deviation. Statistical tests performed are two-way ANOVA with Sidak's correction. Values above bars indicate p-values.

E) γH2A.X expression in control and *Chd1* KO cells across cell cycle stages. Graph shows mean ± SD of median fluorescence intensity (MFI) normalized to negative control cells. N=4 biologically independent experiments. Graphs show mean and standard deviation. Statistical tests performed are two-way ANOVA with Sidak's correction. Values above bars indicate p-values.

Gene name	Primer F	Primer R
rDNA promoter	cctttgaggtccggttcttt	tccaggtccaataggaacagat
28S rRNA	aaatgtggcgtacggaagac	cgtgccggtatttagcctta
neg control	AAGGGGCCTCTGCTTAAAAA	AGAGCTCCATGGCAGGTAGA
rDNA enhancer	tacttctgaggccgagagga	gatccaaagctccagctgac
18S	gtggagcgatttgtctggtt	cgctgagccagtcagtgtag
5.8S	gactcttagcggtggatcactc	gacgctcagacaggcgtag
ITS	gtgtcgttcccgtgtttttc	atcggtatttcgggtgtgag
ETS	cgtcttctcctccgtctcc	gatcccaccgtcggtcac
IGS-1	tcttccgaaggtgcagagtt	tcctcctcctcctcctcttc
IGS-2	cttcccaaatgctgggatta	aaggcagctagggctacaca
IGS-3	cttcccaaatgctgggatta	acaaggcagctagggctaca
IGS-4	ccatctcgtgggcttatgtt	aggcagagatgggaggattt
Usp46	CTCGGCGGCCACTCACCATA	GTAAAGATGGCGGTGCGCGA
Rif1	AAATAAGCGCGAGCCGGGAG	CCACGCGGGAAGTCCGCTC
Rfx2	GGAGTTCGGCAGGGAGTTCCG	TTACCAACGCGGAGCGACAC
Mpp5	GTAAGGGGCGGACCCGCAG	CTGGGAGGGGGTGCGAGC

Supplementary Table 1. List of primers.
