# Supplementary material for: Chd1 protects genome integrity at promoters to sustain hypertranscription in embryonic stem cells
Source: Nat Commun. 2021 Aug 11;12:4859. doi: 10.1038/s41467-021-25088-3 (PMC8357957; doi:10.1038/s41467-021-25088-3)
Supplement: Supplementary file 3 — Description of Additional Supplementary Files [file 41467_2021_25088_MOESM3_ESM.pdf]

Description of Additional Supplementary Files

File Name: Supplementary Data 1
Description: Putative Chd1-interacting proteins identified by IP-mass spectrometry

File Name: Supplementary Data 2
Description: Gene ontology analysis of Chd1-interacting proteins

File Name: Supplementary Data 3
Description: List of DSB-prone genes
