# Supplementary material for: Chd1 protects genome integrity at promoters to sustain hypertranscription in embryonic stem cells
Source: Nat Commun. 2021 Aug 11;12:4859. doi: 10.1038/s41467-021-25088-3 (PMC8357957; doi:10.1038/s41467-021-25088-3)
Supplement: Supplementary file 7 — Reporting Summary [file 41467_2021_25088_MOESM7_ESM.pdf]

nature research

Corresponding author(s): Miguel Ramalho-Santos, Aydan Bulut-Karslioglu

Last updated by author(s): Jun 15, 2021

Reporting Summary

Nature Research wishes to improve the reproducibility of the work that we publish. This form provides structure for consistency and transparency in reporting. For further information on Nature Research policies, see our Editorial Policies and the Editorial Policy Checklist.

Statistics

For all statistical analyses, confirm that the following items are present in the figure legend, table legend, main text, or Methods section.

n/a	Confirmed	
☐	☒	The exact sample size (*n*) for each experimental group/condition, given as a discrete number and unit of measurement
☐	☒	A statement on whether measurements were taken from distinct samples or whether the same sample was measured repeatedly
☐	☒	The statistical test(s) used AND whether they are one- or two-sided<br>*Only common tests should be described solely by name; describe more complex techniques in the Methods section.*
☒	☐	A description of all covariates tested
☐	☒	A description of any assumptions or corrections, such as tests of normality and adjustment for multiple comparisons
☐	☒	A full description of the statistical parameters including central tendency (e.g. means) or other basic estimates (e.g. regression coefficient) AND variation (e.g. standard deviation) or associated estimates of uncertainty (e.g. confidence intervals)
☐	☒	For null hypothesis testing, the test statistic (e.g. *F*, *t*, *r*) with confidence intervals, effect sizes, degrees of freedom and *P* value noted<br>*Give P values as exact values whenever suitable.*
☒	☐	For Bayesian analysis, information on the choice of priors and Markov chain Monte Carlo settings
☒	☐	For hierarchical and complex designs, identification of the appropriate level for tests and full reporting of outcomes
☒	☐	Estimates of effect sizes (e.g. Cohen's *d*, Pearson's *r*), indicating how they were calculated

Our web collection on statistics for biologists contains articles on many of the points above.

Software and code

Policy information about availability of computer code

Data collection

No software was used.

Data analysis

ChIP-seq, DSB-seq, and MNase-seq data processing
ChIP-seq and DSB-seq reads were mapped to the mm9 genome using Bowtie2 with the options --end-to-end --sensitive --score-min L,-1.5,-0.3. Reads with mapping quality smaller than 13 were filtered out. PCR duplicates were removed by keeping at most one mapped read at each genomic position. Biological replicates were then combined. Read coverage profiles were generated using bedtools after extending the mapped reads from 5' to 3' end to 200 bp. Processed MNase-seq data in mouse ES cells were obtained from Voong et al.19 with accession number GSE82127. Center-weighted nucleosome occupancy was calculated from the provided nucleosome center scores as described in Voong et al. and used throughout the paper.

DSB-seq peak calling and annotation
DSB-seq peaks were called using MACS2 with the options --shift 0 --nomodel --extsize 200 -g mm and default q-value cutoff 0.05. 5903 and 183 peaks were identified for Chd1 KO and WT cells, respectively. After filtering out peaks overlapping with blacklisted regions (http://mitra.stanford.edu/kundaje/akundaje/release/blacklists/mm9-mouse/mm9-blacklist.bed.gz), 5671 and 54 peaks were retained for Chd1 KO and WT cells, respectively. The identified peaks were associated with genomic annotations using HOMER annotatePeaks.pl. In particular, 1825 peaks in Chd1 KO cells were mapped around TSSs (-1kb to +100bp) of 1785 genes (DSB-prone genes).

Gene annotations
RefSeq gene annotations were downloaded from UCSC Table Browser. In order to avoid ambiguity, only protein-coding genes with unique TSSs in autosomes were used. Genes whose TSSs +/- 10kb overlap with blacklisted regions were further filtered out. As a result, 14109 genes were retained and used throughout the paper. In Figure 4B, we further required that the gene has a unique expression value provided by Guzman-Ayala et al. (GSE57609), resulting in 7524 genes. In Figure 4D-F, 1107 DSB prone genes (intersection between the 1785 genes identified by HOMER and the 14109 filtered genes) were compared with 13002 (=14109-1107) non-DSB-prone genes. In Figure 4F, the mean

gene length of different isoforms was used for genes with different transcription termination sites (TTS). Gene ontology analysis was performed using the DAVID software.

IP-MS
Data was analysed using Mascot (Matrix Sciences) the parameters were; Uniprot database, taxonomy Mus Musculus, 16403 sequences present, trypsin with up to 1 missed cleavage allowed, variable modification were oxidised methionine, phosphorylated serine, threonine and tyrosine and the peptide tolerance of 0.025 Da and 0.03 Da for MS/MS tolerance. The false discovery rate (FDR) of protein identification was controlled using a target-decoy searching strategy35. The maximum allowed FDR for protein identification was set to 1%. The posterior error probability (PEP) score denotes the probability that the identified peptide is correct.

For gene ontology (GO) and protein network analysis, proteins with fewer than 4 peptides in Chd1 IP were filtered out and only those hits passing the log2FC (Chd1 IP/negative control IP)>2 and PEP score<0.05 were retained. Gene ontology (GO) pathway enrichment analysis was performed using the compareCluster function of clusterProfiler36 (version 3.18.1). The “enrichGO” function for biological process with a significance cutoff p-value < 0.05 and q-value < 0.1 was used. Benjamini-Hochberg method was used to correct for multiple statistical testing (adjusted p-values).

Protein network analysis was performed using the STRING software37 (version 11.0) with the following parameters: Network type: Full STRING network, meaning of network edges: confidence (line thickness indicates the strength of data support), active interaction sources: experiments, textmining, databases, co-expression, neighborhood, gene fusion, co-occurance, minimum required interaction score=0.4.

Version info for used software:
Bowtie (version 2.2.6)
MACS (version 2.1.1)
HOMER (version 4.10.4)
DAVID (version 6.8)
STRING (version 11.0)
ClusterProfiler (version 3.18.1)

For manuscripts utilizing custom algorithms or software that are central to the research but not yet described in published literature, software must be made available to editors and reviewers. We strongly encourage code deposition in a community repository (e.g. GitHub). See the Nature Research guidelines for submitting code & software for further information.

Data

Policy information about availability of data

All manuscripts must include a data availability statement. This statement should provide the following information, where applicable:
- Accession codes, unique identifiers, or web links for publicly available datasets
- A list of figures that have associated raw data
- A description of any restrictions on data availability

ChIP-seq and DSB-seq data have been deposited in Gene Expression Omnibus (GEO) under accession number GSE132137. Mass spectrometry data have been deposited to ProteomeXchange Consortium via the PRIDE34 partner repository with the dataset identifier PXD024604. Both datasets are publicly available.

Field-specific reporting

Please select the one below that is the best fit for your research. If you are not sure, read the appropriate sections before making your selection.

☒ Life sciences ☐ Behavioural & social sciences ☐ Ecological, evolutionary & environmental sciences

For a reference copy of the document with all sections, see nature.com/documents/nr-reporting-summary-flat.pdf

Life sciences study design

All studies must disclose on these points even when the disclosure is negative.

Sample size	No sample size calculation was performed. At least two biological replicates were used for NGS experiments (ChIP-seq, DSB-seq). At least three replicates were used for qPCR experiments. At least 4 embryos per group was used for embryo experiments. The sample sizes of sequencing datasets are in line with current standards and allow statistical analysis. Similarly, IF and qPCR experiments' sample size was estimated based on current standards. At least two replicates were performed for IP-MS and WB experiment to ensure reproducibility with biologically independent samples. IP-MS results are supported by mass spectrometry data.
Data exclusions	No data were excluded
Replication	Experiments were replicated independently at least three times except western blot experiments which were replicated two times. Experiment-specific information is provided in the corresponding legends.<br><br>In specific cases, multiple antibodies were used. All results but phospho-Kap1 subcellular localization data were reproduced. pKap1 immunofluorescence images were thus removed. Antibody batch variations might underlie non-reproducibility of pKap1.
Randomization	Samples were not randomized as phenotype is evident in KO tissues and cells.

Blinding	Investigators were not blinded. Phenotype is evident in KO tissues and cells. The follow-up analysis of sequencing datasets were performed in an unbiased way for all control and experimental samples.

Reporting for specific materials, systems and methods

We require information from authors about some types of materials, experimental systems and methods used in many studies. Here, indicate whether each material, system or method listed is relevant to your study. If you are not sure if a list item applies to your research, read the appropriate section before selecting a response.

Materials & experimental systems

n/a	Involved in the study
☐	☒ Antibodies
☐	☒ Eukaryotic cell lines
☒	☐ Palaeontology and archaeology
☐	☒ Animals and other organisms
☒	☐ Human research participants
☒	☐ Clinical data
☒	☐ Dual use research of concern

Methods

n/a	Involved in the study
☐	☒ ChIP-seq
☐	☒ Flow cytometry
☒	☐ MRI-based neuroimaging

Antibodies

Antibodies used	ChIP: Flag (Sigma, F1804), Kap1 (Abcam, ab22553), phospho S824 Kap1 (Abcam, ab70369), gH2A.X (Abcam, ab2893), H2A.X (Abcam, ab11175), Atm (Genetex, GTX10701).<br>Western blot: Chd1 (Cell Signaling, 4351), Top2b(Santa Cruz Biotechnology, sc-13059), Atm (Genetex, GTX70103 and Abcam, ab78), p-Atm (Thermo, MA1-2020), Kap1 (Abcam, ab22553), Nucleolin (Abcam, ab22758), Polr1a (Cell Signaling, D6S6S), H2A.X (Abcam, ab11175), gH2A.X (Abcam, ab2893), Gapdh (Millipore, MAB-374), Xrcc1 (Santa Cruz Biotechnology, sc-11429), anti-rabbit/mouse/goat secondary antibodies (Jackson Labs, 115-035-062, 111-035-144).<br>IF: Kap1 (Abcam, ab22553), phospho S824 Kap1 (Abcam, ab70369), gH2A.X (Abcam, ab2893), Nucleolin (Abcam, ab22758), Atm (Genetex, GTX70103), Top2b (Santa Cruz Biotechnology, sc-13059), 53BP1 (Abcam, ab175933), and Fibrillarin (Abcam, ab4566).
Validation	Antibody validations were performed by manufacturers and include western blot detection in cell extracts, immunoprecipitation, immunofluorescence or ChIP. Flag antibody was validated in our lab using a wild-type cell line.

Eukaryotic cell lines

Policy information about cell lines

Cell line source(s)	Chd1-Flag knock-in, Chd1fl/del and Chd1del/del ES cell lines were generated in the Ramalho-Santos lab. E14 cell lines was originally acquired from B.Skarnes at Sanger Institute.
Authentication	Cell lines were not authenticated.
Mycoplasma contamination	Cell lines tested negative for mycoplasma.
Commonly misidentified lines (See ICLAC register)	No commonly misidentified cell lines were used in the study.

Animals and other organisms

Policy information about studies involving animals; ARRIVE guidelines recommended for reporting animal research

Laboratory animals	Chd1del/+ females (6- to 12-week-old) and males (6 week- to 6-month-old) (SW background) were used to recover the embryos.
Wild animals	No wild animals were used.
Field-collected samples	No field-collected samples were used.
Ethics oversight	All procedures involving animals were performed in compliance with the protocol approved by the IACUC at UCSF, as part of an AAALAC-accredited care and use program (protocol AN091331-03).

Note that full information on the approval of the study protocol must also be provided in the manuscript.

ChIP-seq

Data deposition

☒ Confirm that both raw and final processed data have been deposited in a public database such as GEO.

☒ Confirm that you have deposited or provided access to graph files (e.g. BED files) for the called peaks.

Data access links<br>*May remain private before publication.*	GSE132137
Files in database submission	Raw fastq files and processed bigwig and bed files are available for DSB-seq as well as Chd1 and RNA PolII ChIP-seq.
Genome browser session<br>(e.g. UCSC)	No longer applicable

Methodology

Replicates	Two biological replicates were performed for DSB-seq and RNA PolII ChIP-seq. No replicates were performed for Chd1 ChIP-seq.
Sequencing depth	All experiments were performed with 50-bp single-end sequencing. Total number of reads and deduplicated uniquely mapped reads are provided below for each experiment.<br>DSB-seq WT rep1: 55783399, 14573142<br>DSB-seq WT rep2: 50645459, 13279910<br>DSB-seq KO rep1: 50835741, 13841441<br>DSB-seq KO rep2: 53148632, 16234625<br>RNA PolII ChIP-seq rep1: 51465826, 29764662<br>RNA PolII ChIP-seq rep2: 42064825, 20129598<br>Chd1 ChIP-seq IP: 102252887, 67003811<br>Chd1 ChIP-seq input: 81324343, 30311678
Antibodies	Following antibodies were used: Flag (Sigma, F1804), Kap1 (Abcam, ab22553), phospho S824 Kap1 (Abcam, ab70369), gH2A.X (Abcam, ab2893), H2A.X (Abcam, ab11175), H1 (Thermo Fisher Scientific, PA128374), Polr1a (Cell Signaling, D6S6S), Atm (Genetex, GTX10701).
Peak calling parameters	For read mapping, we used Bowtie2 with the options --end-to-end --sensitive --score-min L,-1.5,-0.3.<br>For DSB-seq peak calling, we used MACS2 with the options --shift 0 --nomodel --extsize 200 -g mm.
Data quality	To ensure data quality, reads with mapping quality smaller than 13 were filtered out, and PCR duplicates were removed by keeping at most one mapped read at each genomic position. For DSB-seq, 5903 and 183 peaks were identified for Chd1 KO and WT cells, respectively, at the q-value cutoff of 0.05. After filtering out peaks overlapping with blacklisted regions, 5671 and 54 peaks were retained for Chd1 KO and WT cells, respectively.
Software	Bowtie2, MACS2, bedtools.

Flow Cytometry

Plots

Confirm that:

☒ The axis labels state the marker and fluorochrome used (e.g. CD4-FITC).

☒ The axis scales are clearly visible. Include numbers along axes only for bottom left plot of group (a 'group' is an analysis of identical markers).

☒ All plots are contour plots with outliers or pseudocolor plots.

☒ A numerical value for number of cells or percentage (with statistics) is provided.

Methodology

Sample preparation	Overnight cultured ES cells were incubated in 10uM 5-ethynyl-2'-deoxyuridine (EdU) for 1 hour. Cells were harvested, fixed in 4% PFA for 15 minutes, and permeabilized in 0.25% Triton-X 100 in PBS for 5 minutes on ice. Cells were washed with 1% BSA in PBS and incubated in γH2A.X primary antibody (Abcam, ab2893) for 20 minutes at room temperature. Following a second wash, cells were incubated with fluorescence-conjugated secondary antibody (Life Technologies) for 20 minutes at room temperature. Subsequent EdU labeling was conducted according to manufacturer instructions of the Click-iT EdU Alexa Fluor 488 Flow Cytometry Assay Kit ((Life Technologies). SYTOX Blue (Invitrogen) was used to detect DNA content at 1:1000 dilution.
Instrument	Sony MA900 Multi-Application Cell Sorter
Software	FCS Express 7

Cell population abundance	Cell cycle distributions for control and Chd1 KO cells were analyzed based on the gating strategy below.
Gating strategy	S phase: EdU high<br>G0/G1 phase: EdU low, SytoxBlue low<br>G2/M phase: EdU low, SytoxBlue high

☒ Tick this box to confirm that a figure exemplifying the gating strategy is provided in the Supplementary Information.
